# Incidence of and risk factors for nonrespiratory acute organ failure in ICU patients receiving respiratory support: a pilot international cohort study

**DOI:** 10.1186/cc9608

**Published:** 2011-03-11

**Authors:** M Terblanche, A Smith, E Recchia, M Harward, L Gilfeather, D McAuley

**Affiliations:** 1 King's College London, UK; 2 Royal London Hospital, London, UK; 3 St Thomas' Hospital, London, UK; 4 Princess Alexandra Hospital, Brisbane, Australia; 5 Altnagelvin Hospital, Londonderry, UK; 6 QUB, Belfast, UK

## Introduction

Strategies to prevent the progression to nonrespiratory multiorgan failure (nrAOF) in patients receiving invasive or non-invasive ventilation are needed. We performed a pilot international prospective cohort study to determine the incidence of and risk for nrAOF in ICU patients receiving respiratory support.

## Methods

All consecutive ICU admissions to 11 ICUs (UK, Australia and Canada) were screened during the first 24 hours over a 4-week period. Patients receiving positive pressure ventilatory support for at least 1 hour during the first 24 hours were eligible. Those with nrAOF (SOFA 3 to 4), or elective postsurgical patients extubated and ready for discharge within 24 hours after admission, were excluded. Follow up lasted for the first of 14 days after enrolment or ICU discharge.

## Results

In total, 123/766 (16.1%) patients were enrolled. Elective postsurgery ventilation (22.1%) and type I respiratory failure (29.5%) were the most frequent indications for respiratory support. *n *= 49 (39.8%, 95% CI = 31.1 to 48.6%) developed nrAOF after an average 3.7 (SD 1.5) days. The 28-day ICU mortality was 8.1%. In univariate analysis, APACHE II >14.5 (OR = 3.0, 95% CI = 1.2 to 7.1) and nonrespiratory SOFA score >1 (OR = 2.3, 95% CI = 1.1 to 4.7 excluding GCS) were associated (*P *< 0.05) with AOF. See Table 1.

**Table 1:** 

Variable	No AOF	AOF	*P *value
Age	54.3 (19.6)	58.2 (19.6)	0.56
Female	24 (58.5%)	17 (41.5%)	0.007
APACHE II	12.1 (6.7)	17.5 (7.1)	< 0.0001
SOFA	1.52 (1.52)	2.9 (2.5)	0.0002

AOF, acute nonrespiratory organ failure; SOFA, excluding respiratory and GCS.

## Conclusions

Nearly 4/10 developed AOF, but the treatment window is relatively small. APACHE II and baseline SOFA may predict risk. These data inform future trials of preventive strategies but a study with more outcome events is needed to reduce the confidence intervals.

